# NeuroPORTRESS: prevalence, clinical spectrum, and factors associated with neurological involvement in Sjögren’s disease

**DOI:** 10.3389/fimmu.2026.1791369

**Published:** 2026-08-10

**Authors:** Roberto Pereira da Costa, Matilde Bandeira, Manuel Silvério-António, Ana R. Lopes, Filipe Cunha-Santos, Paulo J. Pereira, Diana Belchior Raimundo, Anita Cunha, Cláudia Pinto Oliveira, Ana C. Duarte, João Madruga Dias, Mariana Emília Santos, Maria J. Gonçalves, Ana C. Moniz, Ana I. Maduro, Mariana Luís, Ana Valido, Margarida Oliveira, Luísa Brites, Catarina Tenazinha, Nikita Khmelinskii, Filipe Barcelos, João E. Fonseca, Vasco C. Romão, PORTRESS Reuma.pt Task Force

**Affiliations:** 1 Serviço de Reumatologia, Unidade Local de Saúde Santa Maria, Centro Académico de Medicina de Lisboa (CAML), Lisboa, Portugal; 2 Faculdade de Medicina, Universidade de Lisboa, Centro Académico de Medicina de Lisboa (CAML), Lisboa, Portugal; 3 Unidade de Reumatologia, Unidade Local de Saúde de Viseu Dão-Lafões, Viseu, Portugal; 4 Serviço de Reumatologia, Unidade Local de Saúde da Guarda, Guarda, Portugal; 5 Serviço de Reumatologia, Unidade Local de Saúde de Braga, Braga, Portugal; 6 Serviço de Reumatologia, Unidade Local de Saúde de Loures-Odivelas, Loures, Portugal; 7 Serviço de Reumatologia, Unidade Local de Saúde do Alto Minho, Ponte de Lima, Portugal; 8 Serviço de Reumatologia, Unidade Local de Saúde da Região de Aveiro, Aveiro, Portugal; 9 Serviço de Reumatologia, Unidade Local de Saúde Almada-Seixal, Almada, Portugal; 10 Serviço de Reumatologia, Unidade Local de Saúde do Médio Tejo, Tomar, Portugal; 11 Serviço de Reumatologia, Unidade Local de Saúde de Lisboa Ocidental, Lisboa, Portugal; 12 Serviço de Reumatologia, Hospital CUF Santarém, Santarém, Portugal; 13 Instituto Politécnico de Lisboa, Escola Superior de Tecnologia da Saúde de Lisboa, Lisboa, Portugal; 14 Serviço de Reumatologia, Unidade Local de Saúde de Coimbra, Coimbra, Portugal; 15 Serviço de Reumatologia, Unidade Local de Saúde do Litoral Alentejano, Santiago do Cacém, Portugal; 16 Serviço de Reumatologia, Unidade Local de Saúde da Cova da Beira, Covilhã, Portugal; 17 Serviço de Reumatologia, Unidade Local de Saúde da Região de Leiria, Leiria, Portugal; 18 Serviço de Reumatologia, Unidade Local de Saúde do Algarve, Faro, Portugal; 19 Serviço de Reumatologia, Hospital CUF Descobertas, Lisboa, Portugal; 20 Instituto Português de Reumatologia, Lisboa, Portugal

**Keywords:** central nervous system, independent associations, neurological involvement, peripheral nervous system, Sjogren’s disease

## Abstract

**Objectives:**

Neurological involvement is a heterogeneous complication of Sjögren’s disease (SjD). We aimed to describe its prevalence and spectrum, and identify factors associated with peripheral (PNS) and central nervous system (CNS) involvement.

**Methods:**

We conducted a cross-sectional, multicentre study based on PORTRESS, the Portuguese SjD registry. Demographic and clinical data were compared between groups. Independent associations of overall neurological, PNS, and CNS involvement were identified through logistic regression models using complete-case analysis.

**Results:**

Among 1234 patients, 62 (5.0%) had neurological involvement: 46 (3.7%) PNS, 17 (1.4%) CNS, with one patient having both. Patients with neurological manifestations had higher ESSDAI, more cryoglobulinaemia, purpura/cutaneous vasculitis and monoclonal gammopathy, and higher mortality. Cryoglobulinaemia and higher baseline ESSDAI (excluding neurological domains) were independently associated with neurological involvement. PNS involvement was associated with older age at symptom onset, cryoglobulinaemia, lymphopenia and higher ESSDAI. A sensitivity analysis revealed low C4, persistent salivary gland swelling and purpura/cutaneous vasculitis also as independently associated with PNS involvement. CNS involvement was associated with male sex and younger age at diagnosis.

**Conclusion:**

Neurological involvement in SjD was infrequent. PNS involvement clustered with vasculitic and B-cell-driven features and worse outcomes, whereas CNS manifestations appeared to show a distinct demographic profile, characterised by younger age and male sex. These findings support separate evaluation of PNS and CNS involvement and may help identify patients requiring neurological surveillance. Given the limited number of cases, particularly of CNS involvement, these findings remain exploratory and require confirmation in larger cohorts.

## Highlights

- Neurological involvement in Sjögren’s disease is infrequent but identifies a high-risk systemic phenotype.
- Peripheral neuropathy clusters with older age, and vasculitic, B-cell–driven features.
- Central nervous system involvement shows a distinct profile, associated with male sex and younger age.

## Introduction

Sjögren’s disease (SjD) is a chronic systemic autoimmune disorder traditionally defined by impaired lacrimal and salivary gland function (1). However, SjD is a heterogeneous multisystem disease that extends far beyond glandular involvement. A wide range of extraglandular manifestations may occur, affecting organs such as the lungs, kidneys, skin, joints, and both the peripheral and the central nervous systems (1–6). Up to 71% of patients develop extraglandular manifestations, which are key determinants of SjD long-term prognosis (1–6).

Neurological involvement is among the most challenging systemic manifestations of SjD. Its reported prevalence varies widely, approximating 20% in many cohorts, although values as high as 68% have been described (7–14). Peripheral nervous system (PNS) involvement has been described in approximately 4-23.5% of patients, while central nervous system (CNS) is usually described in fewer than 10%, although higher estimates have also been reported (2–9, 15, 16, 19, 20). PNS manifestations include axonal neuropathy, demyelinating polyneuropathy, ganglionopathy, small fibre neuropathy, mononeuropathy, and peripheral cranial neuropathies, whereas CNS involvement may present as cerebral vasculitis, encephalitis, aseptic meningitis, transverse myelitis, cranial nerve disorders, multiple sclerosis-like syndromes, and neuromyelitis optica spectrum disorders (NMOSD) (7, 17, 18). Autonomic neuropathy is an additional, still poorly understood and frequently underdiagnosed manifestation of SjD (18). Recent consensus efforts aim to better define these presentations and guide management, but substantial unmet needs remain (18).

Overall, the wide variability in reported neurological involvement likely reflects methodological heterogeneity, including differences in case definitions, recruitment settings, and screening strategies, rather than true biological variation alone. In particular, the requirement for objective confirmation of neurological involvement may substantially influence prevalence estimates.

The pathogenesis of neurological involvement in SjD also remains incompletely understood and is likely heterogeneous. Several mechanisms have been proposed, including vasculitic and ischaemic injury, lymphocytic infiltration of neural tissues, and antibody-mediated processes (18, 21).

Many patients may present with neurological manifestations before developing sicca symptoms (21). In such cases, glandular involvement may be minimal or absent. As a result, many patients do not meet classification criteria at disease onset and may be overlooked. This diagnostic gap likely contributes to their under-representation in clinical cohorts and research studies, further limiting our understanding of the prevalence, spectrum, and factors associated with neurological involvement in SjD.

Despite the clinical significance of neurological manifestations, including their substantial weighting in the EULAR Sjögren’s syndrome disease activity (ESSDAI) score (17), factors associated with neurological involvement remain insufficiently characterised (18). Although previous studies have provided important insights into neurological involvement in SjD, many have been restricted to patients fulfilling established classification criteria. This may under-represent clinically diagnosed patients with early or predominantly extraglandular disease. In addition, several studies have focused specifically on either PNS or CNS manifestations or analysed neurological involvement as a single composite outcome (4–13, 19, 20, 22–26). Therefore, data from large real-world cohorts including clinically diagnosed patients and evaluating both PNS and CNS manifestations within the same population, while also exploring their potentially distinct associated factors, remain limited.

The aims of this study were to describe the frequency and characteristics of neurological involvement in a nationwide SjD cohort, and to identify demographic and clinical factors associated with overall, PNS and CNS involvement.

## Methods

### Study design and patient selection

This multicentre cohort study included patients registered in PORTRESS, the SjD module of the nationwide Rheumatic Diseases Portuguese registry - Reuma.pt (3), based on a retrospective analysis of prospectively collected data. Patients recorded with a diagnosis of SjD in PORTRESS up to November 2023 and with available information on neurological involvement were eligible for inclusion. Patients with missing information on neurological involvement were excluded. (**Supplementary Figure 1**) Demographic and clinical data were collected, including fulfillment of the 2002 American-European Consensus Group (AECG) classification criteria and of the 2016 American College of Rheumatology/European League Against Rheumatism (ACR/EULAR) classification criteria for SjD (27, 28). All patients were followed in a Rheumatology department.

### Neurological assessment and definitions

Neurological involvement was determined by the attending physician and required compatible clinical manifestations supported by appropriate diagnostic investigations, including magnetic resonance imaging (MRI) for CNS syndromes, cerebrospinal fluid analysis when appropriate, nerve conduction studies for peripheral neuropathies, and disease-specific antibodies when clinically indicated (e.g., aquaporin-4 for NMOSD). Alternative causes of neurological manifestations were excluded based on clinical evaluation and complementary investigations. The electronic records of the patients classified as having neurological involvement were reviewed in order to confirm the diagnosis, categorise PNS and/or CNS involvement, and collect supporting data. Patients with only autonomic involvement were not considered, since most centres lack the capability to perform comprehensive autonomic testing, making definitive confirmation or exclusion unreliable. Baseline was defined as the time of the first available registry entry, corresponding to the initial recorded assessment in Reuma.pt, at which clinical and disease activity data, including ESSDAI, were collected, and regardless of the date of neurological involvement.

### Statistical analysis

Descriptive statistics are presented as mean ± standard deviation for normally distributed variables, as median (interquartile range - IQR) for non-normally distributed variables, and as absolute and relative frequencies for categorical variables. Categorical variables were compared using the chi-square test or Fisher’s exact test, as appropriate. Continuous variables were compared using Student’s t-test or Mann-Whitney test, according to data distribution. No formal correction for multiple comparisons was applied.

Multivariable analysis with binomial logistic regression with backward selection, informed by clinical relevance and prior evidence, was performed to identify factors associated with neurological (PNS and/or CNS), PNS (versus no-PNS) and CNS (versus no-CNS) involvement, after appropriate collinearity analysis. CNS-only patients were included in the no-PNS comparator group, and PNS-only patients were included in the no-CNS comparator group. The single patient with both PNS and CNS involvement was included in both subgroup analyses and excluded from the direct comparison between PNS and CNS subgroups.

Analyses were performed using available data for each variable. No imputation was performed. The proportion of missing data for each variable was reported and categorised as <10%, 10-25%, 25-50% and >50%. (**Tables 1**–**4**) In multivariable models, analyses were restricted to patients with complete data for all variables included in each model; consequently the effective sample size differed across models according to data availability. Candidate variables and the effective sample size for each multivariable model are reported in Supplementary **Tables 1**-**3**. Given the real-world nature of the registry and the inclusion of clinically diagnosed cases not fulfilling classification criteria, sensitivity analyses with logistic regression models restricted to patients fulfilling at least one set of SjD classification criteria were performed to assess the robustness of the findings.

**Table 1:** Comparison of demographic and clinical characteristics of SjD patients with neurological (PNS and/or CNS) involvement and without neurological involvement.

	SjD patients (total) n=1234	SjD patients with neurological involvement n=62	SjD patients without neurological involvement n=1172	*p* value
Female sex, *n* (%)	1162 (94.2)	54 (87.1)	1108 (94.5)	**0.024**
Caucasian*^φ^, *n* (%)	968 (92.9)	50 (94.3)	918 (92.8)	1.000
Age at inclusion*^θ^, *years*±SD	61.8±14.3	63.1±16.1	61.7±14.2	0.467
Age at symptom onset*^φ^, *years*±SD	49.4±14.8	50.3±14.8	47.8±14.8	0.228
Age at diagnosis*^θ^, *years*±SD	52.8±14.6	53.6±15.0	52.8±14.6	0.675
Disease duration*^φ^, *years* (IQR)	11.0 (11.2)	11.2 (11.4)	11.0 (11.2)	0.412
Classification criteria				
AECG 2002*^φ^, *n (%)*	700 (63.8)	35 (58.3)	665 (64.1)	0.364
ACR/EULAR 2016*^θ^, *n (%)*	717 (60.1)	39 (62.9)	678 (59.9)	0.643
Immune profile				
ANA*^θ^, *n (%)*	1015 (90.5)	54 (88.5)	961 (90.7)	0.579
anti-SSA*^θ^, *n (%)*	972 (80.5)	48 (78.7)	924 (80.6)	0.709
anti-SSB/anti-La*^θ^, *n (%)*	513 (44.2)	23 (38.3)	490 (44.5)	0.349
Rheumatoid factor*^φ^, *n (%)*	512 (48.1)	25 (46.3)	487 (48.2)	0.783
Minor salivary gland biopsy findings				
Focus score≥1*^∇^, *n (%)*	429 (48.7)	24 (55.8)	405 (48.3)	0.338
Focus score*^◻^, *score* (IQR)	1.0 (1.54)	1.2 (2.5)	1.0 (1.5)	0.837
Ectopic lymphoid structures on salivary glands*^∇^, *n (%)*	45 (6.4)	3 (8.8)	42 (6.3)	0.476
Clinical and laboratory characteristics associated with B-cell activity and lymphoproliferative burden				
Cryoglobulinaemia*^∇^, *n (%)*	50 (6.5)	7 (15.2)	43 (6.0)	**0.024**
Hypergammaglobulinaemia*^φ^, *n (%)*	518 (48.3)	28 (47.5)	490 (48.4)	0.891
Monoclonal gammopathy*^φ^, *n (%)*	60 (5.7)	7 (12.3)	53 (5.3)	**0.039**
Low C3*^φ^, *n (%)*	190 (17.7)	11 (18.6)	179 (17.6)	0.838
Low C4*^φ^, *n (%)*	98 (9.1)	9 (15.3)	89 (8.8)	0.091
Lymphopenia*^φ^, *n (%)*	223 (20.3)	16 (26.7)	207 (20.0)	0.211
Persistent salivary gland swelling*^φ^, *n (%)*	84 (7.6)	8 (13.6)	76 (7.3)	0.123
Purpura/cutaneous vasculitis*^φ^, *n (%)*	67 (6.1)	9 (15.5)	58 (5.6)	**0.007**
Lymphoma				
Lymphoma*^∇^, *n (%)*	19 (2.3)	1 (2.4)	18 (2.3)	1.000
Non-Hodgkin lymphoma*^∇^, *n (%)*	3 (0.4)	0	3 (0.4)	1.000
Baseline evaluation				
ESSDAI*^φ^, 0-123 (IQR)	2.0 (4.0)	6.0 (12.0)	2.0 (4.0)	**<0.001**
ESSDAI without neurological domains*^φ^, 0-93 (IQR)	2.0 (4.0)	4.0 (7.0)	2.0 (4.0)	**0.002**
ESSPRI*^◻^, 0-10 (IQR)	5.7 (4.7)	5.8 (5.1)	5.5 (4.7)	0.457
Involvements (definitions according to ESSDAI score)				
Constitutional*^θ^, *n (%)*	224 (18.2)	14 (23.0)	210 (18.0)	0.329
Lymphadenopathic*^θ^, *n (%)*	137 (11.1)	9 (14.8)	128 (10.9)	0.356
Glandular*^θ^, *n (%)*	382 (31.0)	22 (36.1)	360 (30.8)	0.383
Articular*^θ^, *n (%)*	531 (43.1)	30 (49.2)	501 (42.8)	0.328
Cutaneous*^θ^, *n (%)*	219 (17.8)	18 (29.5)	201 (17.2)	**0.014**
Pulmonary*^θ^, *n (%)*	101 (8.2)	11 (18.0)	90 (7.7)	**0.004**
Renal*^θ^, *n (%)*	33 (2.7)	2 (3.3)	31 (2.7)	0.674
Muscular*^θ^, *n (%)*	18 (1.5)	0	18 (1.5)	1.000
PNS, *n (%)*	46 (3.7)	46 (74.2)	0	**<0.001**
CNS, *n (%)*	17 (1.4)	17 (27.4)	0	**<0.001**
Haematological*^θ^, *n (%)*	418 (34.0)	24 (40.7)	394 (33.7)	0.268
Biological*^θ^, *n (%)*	632 (51.5)	37 (61.7)	595 (50.9)	0.105
Treatment (ever)				
Corticosteroids*^∇^, *n (%)*	335 (39.0)	34 (64.2)	301 (37.3)	**<0.001**
Hydroxychloroquine*^∇^, *n (%)*	673 (78.3)	34 (64.2)	639 (79.3)	**0.010**
Pilocarpine*^∇^, *n (%)*	229 (26.7)	11 (20.8)	218 (27.0)	0.316
Methotrexate*^∇^, *n (%)*	144 (16.8)	10 (18.9)	134 (16.6)	0.672
Azathioprine*^∇^, *n (%)*	97 (11.3)	18 (34.0)	79 (9.8)	**<0.001**
Leflunomide*^∇^, *n (%)*	33 (3.8)	2 (3.8)	31 (3.8)	1.000
Mycophenolate mofetil*^∇^, *n (%)*	27 (3.1)	3 (5.7)	24 (3.0)	0.229
Rituximab*^∇^, *n (%)*	38 (4.4)	10 (18.9)	28 (3.5)	**<0.001**
Intravenous immunoglobulin*^∇^, *n (%)*	6 (0.7)	2 (3.8)	4 (0.5)	**0.048**
Cyclophosphamide*^∇^, *n (%)*	6 (0.7)	5 (9.4)	1 (0.1)	**<0.001**
Death				
Death*^∇^, *n (%)*	7 (1.0)	3 (6.1)	4 (0.6)	**0.010**

Categorical variables are presented as frequency (percentage), and continuous variables as mean±standard deviation (SD) or median (interquartile range - IQR) for variables with skewed distributions. NA - not applicable.

θMissing data <10%; φ Missing data 10-25%; ∇ Missing data 25-50%; ◻Missing data >50%.

*SjD patients - Race: n=1042; Age at inclusion: n=1231; Age at symptom onset: n=950; Age at diagnosis: n=1122; Disease duration: n=950; AECG 2002: n=1097; ACR/EULAR 2016: n=1193; ANA: n=1121; anti-SSA: n=1207; anti-SSB: n=1161; Rheumatoid factor: n=1064; Focus score≥1: n=881; Focus score: n=270; Ectopic lymphoid structures on salivary glands: n=698; Cryoglobulinaemia: n=767; Hypergammaglobulinaemia: n=1072; Monoclonal gammopathy: n=1049; Low C3: n=1076; Low C4: n=1076; Lymphopenia: n=1096; Persistent salivary gland swelling: n=1102; Purpura/cutaneous vasculitis: n=1095; Lymphoma: n=816; Non-Hodgkin lymphoma: n=816; ESSDAI: n=985; ESSDAI without neurological domains: n=985; ESSPRI: n=582; Constitutional: n=1228; Lymphadenopathic: n=1231; Glandular: n=1231; Articular: n=1231; Cutaneous: n=1229; Pulmonary: n=1229; Renal: n=1229; Muscular: n=1230; Haematological: n=1229; Biological: n=1228; Corticosteroids: n=859; Hydroxychloroquine: n=859; Pilocarpine: n=859; Methotrexate: n=859; Azathioprine: n=859; Leflunomide: n=859; Mycophenolate mofetil: n=859; Rituximab: n=859; Intravenous immunoglobulin: n=859; Cyclophosphamide: n=859; Death: n=688.

**SjD patients with neurological involvement - Race: n=53; Age at inclusion: n=60; Age at symptom onset: n=54; Age at diagnosis: n=59; Disease duration: n=54; AECG 2002: n=60; ANA: n=61; anti-SSA: n=61; anti-SSB: n=60; Rheumatoid factor: n=54; Focus score≥1: n=43; Focus score: n=23; Ectopic lymphoid structures on salivary glands: n=34; Cryoglobulinaemia: n=46; Hypergammaglobulinaemia: n=59; Monoclonal gammopathy: n=57; Low C3: n=59; Low C4: n=59; Lymphopenia: n=60; Persistent salivary gland swelling: n=59; Purpura/cutaneous vasculitis: n=58; Lymphoma: n=42; Non-Hodgkin lymphoma: n=42; ESSDAI: n=55; ESSDAI without neurological domains: n=55; ESSPRI: n=38; Constitutional: n=61; Lymphadenopathic: n=61; Glandular: n=61; Articular: n=61; Cutaneous: n=61; Pulmonary: n=61; Renal: n=60; Muscular: n=60; Haematological: n=59; Biological: n=60; Corticosteroids: n=53; Hydroxychloroquine: n=53; Pilocarpine: n=53; Methotrexate: n=53; Azathioprine: n=53; Leflunomide: n=53; Mycophenolate mofetil: n=53; Rituximab: n=53; Intravenous immunoglobulin: n=53; Cyclophosphamide: n=53; Death: n=49.

***SjD patients without neurological involvement - Race: n=989; Age at inclusion: n=1171; Age at symptom onset: n=896; Age at diagnosis: n=1063; Disease duration: n=896; AECG 2002: n=1037; ACR/EULAR 2016: N=1131; ANA: n=1060; anti-SSA: n=1146; anti-SSB: n=1101; Rheumatoid factor: n=1010; Focus score≥1: n=838; Focus score: n=247; Ectopic lymphoid structures on salivary glands: n=664; Cryoglobulinaemia: n=721; Hypergammaglobulinaemia: n=1013; Monoclonal gammopathy: n=992; Low C3: n=1017; Low C4: n=1017; Lymphopenia: n=1036; Persistent salivary gland swelling: n=1043; Purpura/cutaneous vasculitis: n=1037; Lymphoma: n=774; Non-Hodgkin lymphoma: n=774; ESSDAI: n=930; ESSDAI without neurological domains: n=930; ESSPRI: n=544; Constitutional: n=1167; Lymphadenopathic: n=1170; Glandular: n=1170; Articular: n=1170; Cutaneous: n=1168; Pulmonary: n=1168; Renal: n=1169; Muscular: n=1170; Haematological: n=1170; Biological: n=1168; Corticosteroids: n=806; Hydroxychloroquine: n=806; Pilocarpine: n=806; Methotrexate: n=806; Azathioprine: n=806; Leflunomide: n=806; Mycophenolate mofetil: n=806; Rituximab: n=806; Intravenous immunoglobulin: n=806; Cyclophosphamide: n=806; Death: n=639. Bold values indicate statistical significance (p<0.05).

**Table 2:** Comparison of demographic and clinical characteristics of SjD patients with and without PNS involvement.

	SjD patients with PNS involvement n=46	SjD patients without PNS involvement n=1188	*p* value
Female sex, *n* (%)	41 (89.1)	1121 (94.4)	0.185
Caucasian*^φ^, *n* (%)	38 (100)	930 (92.6)	0.104
Age at inclusion*^θ^, *years*±SD	67.8±13.1	61.6±14.3	**0.004**
Age at symptom onset*^φ^, *years*±SD	53.1±13.7	47.7±14.8	**0.024**
Age at diagnosis*^θ^, *years*±SD	57.2±13.0	52.7±14.6	**0.042**
Disease duration*^φ^, *years* (IQR)	12.0 (13.0)	11.0 (10.5)	0.118
Classification criteria			
AECG 2002*^φ^, *n (%)*	26 (59.1)	674 (64.0)	0.506
ACR/EULAR 2016*^θ^, *n (%)*	28 (60.9)	689 (60.1)	0.914
Immune profile			
ANA*^θ^, *n (%)*	40 (88.9)	975 (90.6)	0.607
anti-SSA*^θ^, *n (%)*	32 (71.1)	940 (80.9)	0.104
anti-SSB*^θ^, *n (%)*	17 (38.6)	496 (44.4)	0.450
Rheumatoid factor*^φ^, *n (%)*	19 (48.7)	493 (48.1)	0.939
Minor salivary gland biopsy findings			
Focus score≥1*^∇^, *n (%)*	19 (59.4)	410 (48.3)	0.218
Focus score*^◻^, *score* (IQR)	1.6 (2.6)	1.0 (1.5)	0.599
Ectopic lymphoid structures on salivary glands*^∇^, *n (%)*	2 (8.0)	43 (6.4)	0.672
Clinical and laboratory characteristics associated with B-cell activity and lymphoproliferative burden			
Cryoglobulinaemia*^∇^, *n (%)*	7 (20.0)	43 (5.9)	**0.005**
Hypergammaglobulinaemia*^φ^, *n (%)*	19 (43.2)	499 (48.5)	0.486
Monoclonal gammopathy*^φ^, *n (%)*	5 (11.9)	55 (5.5)	0.086
Low C3*^φ^, *n (%)*	10 (23.3)	180 (17.4)	0.326
Low C4*^φ^, *n (%)*	9 (20.9)	89 (8.6)	**0.012**
Lymphopenia*^φ^, *n (%)*	15 (34.1)	208 (19.8)	**0.021**
Persistent salivary gland swelling*^φ^, *n (%)*	8 (18.6)	76 (7.2)	**0.013**
Purpura/cutaneous vasculitis*^φ^, *n (%)*	8 (19.0)	59 (5.6)	**0.003**
Lymphoma			
Lymphoma*^∇^, *n (%)*	1 (3.3)	18 (2.3)	0.513
Non-Hodgkin lymphoma*^∇^, *n (%)*	0	3 (0.4)	1.000
Baseline evaluation			
ESSDAI*^φ^, 0-123 (IQR)	9.0 (11.8)	2.0 (4.0)	**<0.001**
ESSDAI without neurological domains*^φ^, 0-93 (IQR)	4.0 (8.0)	2.0 (4.0)	**0.003**
ESSPRI*^◻^, 0-10 (IQR)	5.8 (3.9)	5.5 (4.7)	0.196
Involvements (definitions according to ESSDAI score)			
Constitutional*^θ^, *n (%)*	11 (24.4)	213 (18.0)	0.272
Lymphadenopathic*^θ^, *n (%)*	5 (11.1)	132 (11.1)	0.997
Glandular*^θ^, *n (%)*	17 (37.8)	365 (30.8)	0.319
Articular*^θ^, *n (%)*	24 (53.3)	507 (42.7)	0.159
Cutaneous*^θ^, *n (%)*	13 (28.9)	206 (17.4)	**0.048**
Pulmonary*^θ^, *n (%)*	10 (22.2)	91 (7.7)	**0.003**
Renal*^θ^, *n (%)*	0	33 (2.8)	0.628
Muscular*^θ^, *n (%)*	0	18 (1.5)	1.000
PNS, *n (%)*	46 (100)	0	NA
CNS, *n (%)*	1 (2.2)	16 (1.3)	0.478
Haematological*^θ^, *n (%)*	17 (39.5)	401 (33.8)	0.436
Biological*^θ^, *n (%)*	27 (61.4)	605 (51.1)	0.181
Treatment (ever)			
Corticosteroids*^∇^, *n (%)*	25 (67.6)	310 (37.7)	**<0.001**
Hydroxychloroquine*^∇^, *n (%)*	25 (67.6)	648 (78.8)	0.104
Pilocarpine*^∇^, *n (%)*	10 (27.0)	219 (26.6)	0.959
Methotrexate*^∇^, *n (%)*	7 (18.9)	137 (16.7)	0.720
Azathioprine*^∇^, *n (%)*	13 (35.1)	84 (10.2)	**<0.001**
Leflunomide*^∇^, *n (%)*	2 (5.4)	31 (3.8)	0.649
Mycophenolate mofetil*^∇^, *n (%)*	3 (8.1)	24 (2.9)	0.106
Rituximab*^∇^, *n (%)*	6 (16.2)	32 (3.9)	**0.004**
Intravenous immunoglobulin*^∇^, *n (%)*	2 (5.4)	4 (0.5)	**0.024**
Cyclophosphamide*^∇^, *n (%)*	4 (10.8)	2 (0.2)	**<0.001**
Death			
Death*^∇^, *n (%)*	3 (8.8)	4 (0.6)	**0.003**

Categorical variables are presented as frequency (percentage), and continuous variables as mean±standard deviation (SD) or median (interquartile range - IQR) for variables with skewed distributions. NA - not applicable.

θMissing data <10%; φ Missing data 10-25%; ∇ Missing data 25-50%; ◻Missing data >50%.

*SjD patients with PNS involvement - Race: n=38; Age at inclusion: n=44; Age at symptom onset: n=41; Age at diagnosis: n=45; Disease duration: n=41; AECG 2002: n=44; ANA: n=45; anti-SSA: n=45; anti-SSB: n=44; Rheumatoid factor: n=39; Focus score≥1: n=32; Focus score: n=15; Ectopic lymphoid structures on salivary glands: n=25; Cryoglobulinaemia: n=35; Hypergammaglobulinaemia: n=44; Monoclonal gammopathy: n=42; Low C3: n=43; Low C4: n=43; Lymphopenia: n=44; Persistent salivary gland swelling: n=43; Purpura/cutaneous vasculitis: n=42; Lymphoma: n=30; Non-Hodgkin lymphoma: n=30; ESSDAI: n=40; ESSDAI without neurological domains: n=40; ESSPRI: n=26; Constitutional: n=45; Lymphadenopathic: n=45; Glandular: n=45; Articular: n=45; Cutaneous: n=45; Pulmonary: n=45; Renal: n=44; Muscular: n=44; Haematological: n=43; Biological: n=44; Corticosteroids: n=37; Hydroxychloroquine: n=37; Pilocarpine: n=37; Methotrexate: n=37; Azathioprine: n=37; Leflunomide: n=37; Mycophenolate mofetil: n=37; Rituximab: n=37; Intravenous immunoglobulin: n=37; Cyclophosphamide: n=37; Death: n=34.

**SjD patients without PNS involvement - Race: n=1004; Age at inclusion: n=1187; Age at symptom onset: n=909; Age at diagnosis: n=1077; Disease duration: n=909; AECG 2002: n=1053; ACR/EULAR 2016: N=1147; ANA: n=1076; anti-SSA: n=1162; anti-SSB: n=1117; Rheumatoid factor: n=1025; Focus score≥1: n=849; Focus score: n=255; Ectopic lymphoid structures on salivary glands: n=673; Cryoglobulinaemia: n=732; Hypergammaglobulinaemia: n=1028; Monoclonal gammopathy: n=1007; Low C3: n=1033; Low C4: n=1033; Lymphopenia: n=1052; Persistent salivary gland swelling: n=1059; Purpura/cutaneous vasculitis: n=1053; Lymphoma: n=786; Non-Hodgkin lymphoma: n=786; ESSDAI: n=945; ESSDAI without neurological domains: n=945; ESSPRI: n=556; Constitutional: n=1183; Lymphadenopathic: n=1186; Glandular: n=1186; Articular: n=1186; Cutaneous: n=1184; Pulmonary: n=1184; Renal: n=1185; Muscular: n=1186; Haematological: n=1186; Biological: n=1184; Corticosteroids: n=822; Hydroxychloroquine: n=822; Pilocarpine: n=822; Methotrexate: n=822; Azathioprine: n=822; Leflunomide: n=822; Mycophenolate mofetil: n=822; Rituximab: n=822; Intravenous immunoglobulin: n=822; Cyclophosphamide: n=822; Death: n=654. Bold values indicate statistical significance (p<0.05).

**Table 3:** Comparison of demographic and clinical characteristics of SjD patients with and without CNS involvement.

	SjD patients with CNS involvement n=17	SjD patients without CNS involvement n=1217	*p* value
Female sex, *n* (%)	14 (82.4)	1148 (94.3)	0.072
Caucasian*^φ^, *n* (%)	13 (81.3)	955 (93.1)	0.099
Age at inclusion*^θ^, *years*±SD	51.5±17.1	62.0±14.2	**0.003**
Age at symptom onset*^φ^, *years*±SD	43.2±15.7	48.0±14.7	0.224
Age at diagnosis*^φ^, *years*±SD	43.7±16.4	53.0±14.5	**0.014**
Disease duration*^φ^, *years* (IQR)	8.6 (8.5)	11.0 (11.1)	0.273
Classification criteria			
AECG 2002*^φ^, *n (%)*	9 (52.9)	691 (64.0)	0.347
ACR/EULAR 2016*^θ^, *n (%)*	12 (70.6)	705 (59.9)	0.374
Immune profile			
ANA*^θ^, *n (%)*	15 (88.2)	1000 (90.6)	0.671
anti-SSA*^θ^, *n (%)*	16 (94.1)	956 (80.3)	0.221
anti-SSB*^θ^, *n (%)*	6 (35.3)	507 (44.3)	0.457
Rheumatoid factor*^φ^, *n (%)*	6 (40.0)	506 (48.2)	0.526
Minor salivary gland biopsy findings			
Focus score≥1*^∇^, *n (%)*	6 (50.0)	423 (48.7)	0.927
Focus score*^◻^, *score* (IQR)	0.9 (1.3)	1.0 (1.5)	0.294
Ectopic lymphoid structures on salivary glands*^∇^, *n (%)*	1 (10.0)	44 (6.4)	0.489
Clinical and laboratory characteristics associated with B-cell activity and lymphoproliferative burden			
Cryoglobulinaemia*^∇^, *n (%)*	0	50 (6.6)	1.000
Hypergammaglobulinaemia*^φ^, *n (%)*	9 (56.3)	509 (48.2)	0.523
Monoclonal gammopathy*^φ^, *n (%)*	2 (12.5)	58 (5.6)	0.232
Low C3*^φ^, *n (%)*	1 (5.9)	189 (17.8)	0.334
Low C4*^φ^, *n (%)*	0	98 (9.3)	0.391
Lymphopenia*^φ^, *n (%)*	1 (5.9)	222 (20.6)	0.221
Persistent salivary gland swelling*^φ^, *n (%)*	0	84 (7.7)	0.634
Purpura/cutaneous vasculitis*^φ^, *n (%)*	1 (5.9)	66 (6.1)	1.000
Lymphoma			
Lymphoma*^∇^, *n (%)*	0	19 (2.4)	1.000
Non-Hodgkin lymphoma*^∇^, *n (%)*	0	3 (0.4)	1.000
Baseline evaluation			
ESSDAI*^φ^, 0-123 (IQR)	4.5 (12.0)	2.0 (4.0)	**0.007**
ESSDAI without neurological domains*^φ^, 0-93 (IQR)	2.5 (5.0)	2.0 (4.0)	0.550
ESSPRI*^◻^, 0-10 (IQR)	4.5 (6.4)	5.7 (4.7)	0.558
Involvements (definitions according to ESSDAI score)			
Constitutional*^θ^, *n (%)*	3 (17.6)	221 (18.2)	1.000
Lymphadenopathic*^θ^, *n (%)*	4 (23.5)	133 (11.0)	0.111
Glandular*^θ^, *n (%)*	5 (29.4)	377 (31.1)	0.884
Articular*^θ^, *n (%)*	6 (35.3)	525 (43.2)	0.511
Cutaneous*^θ^, *n (%)*	5 (29.4)	214 (17.7)	0.205
Pulmonary*^θ^, *n (%)*	2 (11.8)	99 (8.2)	0.644
Renal*^θ^, *n (%)*	2 (11.8)	31 (2.6)	0.074
Muscular*^θ^, *n (%)*	0	18 (1.5)	1.000
PNS, *n (%)*	1 (5.9)	45 (3.7)	0.478
CNS, *n (%)*	17 (100)	0	NA
Haematological*^θ^, *n (%)*	7 (41.2)	411 (33.9)	0.530
Biological*^θ^, *n (%)*	10 (58.8)	622 (51.4)	0.541
Treatment (ever)			
Corticosteroids*^∇^, *n (%)*	10 (58.8)	325 (38.6)	0.091
Hydroxychloroquine*^∇^, *n (%)*	10 (58.8)	663 (78.7)	0.069
Pilocarpine*^∇^, *n (%)*	1 (5.9)	228 (27.1)	0.054
Methotrexate*^∇^, *n (%)*	3 (17.6)	141 (16.7)	1.000
Azathioprine*^∇^, *n (%)*	6 (35.3)	91 (10.8)	**0.008**
Leflunomide*^∇^, *n (%)*	0	33 (3.9)	1.000
Mycophenolate mofetil*^∇^, *n (%)*	0	27 (3.2)	1.000
Rituximab*^∇^, *n (%)*	4 (23.5)	34 (4.0)	**0.005**
Intravenous immunoglobulin*^∇^, *n (%)*	0	6 (0.7)	1.000
Cyclophosphamide*^∇^, *n (%)*	1 (5.9)	5 (0.6)	0.113
Death			
Death*^∇^, *n (%)*	0	7 (1.0)	1.000

Categorical variables are presented as frequency (percentage), and continuous variables as mean±standard deviation (SD) or median (interquartile range - IQR) for variables with skewed distributions. NA - not applicable.

θMissing data <10%; φ Missing data 10-25%; ∇ Missing data 25-50%; ◻Missing data >50%.

*SjD patients with CNS involvement - Race: n=16; Age at symptom onset: n=14; Age at diagnosis: n=15; Disease duration: n=14; Rheumatoid factor: n=15; Focus score≥1: n=12; Focus score: n=8; Ectopic lymphoid structures on salivary glands: n=10; Cryoglobulinaemia: n=12; Hypergammaglobulinaemia: n=16; Monoclonal gammopathy: n=16; Lymphoma: n=12; Non-Hodgkin lymphoma: n=12; ESSDAI: n=16; ESSDAI without neurological domains: n=16; ESSPRI: n=12; Death: n=15.

**SjD patients without CNS involvement - Race: n=1026; Age at inclusion: n=1214; Age at symptom onset: n=936; Age at diagnosis: n=1107; Disease duration: n=936; AECG 2002: n=1080; ACR/EULAR 2016: n=1176; ANA: n=1104; anti-SSA: n=1190; anti-SSB: n=1144; Rheumatoid factor: n=1049; Focus score≥1: n=869; Focus score: n=262; Ectopic lymphoid structures on salivary glands: n=688; Cryoglobulinaemia: n=755; Hypergammaglobulinaemia: n=1056; Monoclonal gammopathy: n=1033; Low C3: n=1059; Low C4: n=1059; Lymphopenia: n=1077; Persistent salivary gland swelling: n=1085; Purpura/cutaneous vasculitis: n=1078; Lymphoma: n=804; Non-Hodgkin lymphoma: n=804; ESSDAI: n=969; ESSDAI without neurological domains: n=969; ESSPRI: n=570; Constitutional: n=1211; Lymphadenopathic: n=1214; Glandular: n=1214; Articular: n=1214; Cutaneous: n=1212; Pulmonary: n=1212; Renal: n=1212; Muscular: n=1213; Haematological: n=1212; Biological: n=1211; Corticosteroids: n=842; Hydroxychloroquine: n=842; Pilocarpine: n=842; Methotrexate: n=842; Azathioprine: n=842; Leflunomide: n=842; Mycophenolate mofetil: n=842; Rituximab: n=842; Intravenous immunoglobulin: n=842; Cyclophosphamide: n=842; Death: n=673. Bold values indicate statistical significance (p<0.05).

**Table 4:** Comparison of demographic and clinical characteristics of SjD patients with CNS involvement and patients with PNS involvement.

	SjD patients with CNS involvement n=16	SjD patients with PNS involvement n=45	*p* value
Female sex, *n* (%)	13 (81.3)	40 (88.9)	0.422
Caucasian*^φ^, *n* (%)	12 (80.0)	37 (100)	**0.021**
Age at inclusion*^θ^, *years*±SD	52.1±13.2	64.7±11.8	**<0.001**
Age at symptom onset*^φ^, *years*±SD	42.0±10.6	56.6±11.7	**0.017**
Age at diagnosis*^φ^, *years*±SD	43.4±10.3	59.9±12.8	**<0.001**
Disease duration*^φ^, *years* (IQR)	6.0 (9.0)	8.0 (1.9)	0.086
Classification criteria			
AECG 2002*^θ^, *n (%)*	9 (56.3)	26 (60.5)	0.770
ACR/EULAR 2016, *n (%)*	11 (68.8)	27 (60.0)	0.535
Immune profile			
ANA*^θ^, *n (%)*	14 (87.5)	39 (88.6)	1.000
anti-SSA*^θ^, *n (%)*	16 (100)	32 (72.7)	**0.025**
anti-SSB*^θ^, *n (%)*	6 (37.5)	17 (39.5)	0.887
Rheumatoid factor*^φ^, *n (%)*	6 (40.0)	19 (48.7)	0.565
Minor salivary gland biopsy findings			
Focus score≥1*^∇^, *n (%)*	5 (45.5)	18 (54.5)	0.601
Focus score*^◻^, *score* (IQR)	1.0 (1.3)	1.4 (2.5)	0.307
Ectopic lymphoid structures on salivary glands*^∇^, *n (%)*	1 (11.1)	2 (8.3)	1.000
Clinical and laboratory characteristics associated with B-cell activity and lymphoproliferative burden			
Cryoglobulinaemia*^∇^, *n (%)*	0	7 (20.6)	0.168
Hypergammaglobulinaemia*^θ^, *n (%)*	9 (60.0)	19 (46.3)	0.365
Monoclonal gammopathy*^θ^, *n (%)*	2 (13.3)	5 (12.2)	1.000
Low C3*^θ^, *n (%)*	1 (6.3)	10 (23.8)	0.259
Low C4*^θ^, *n (%)*	0	9 (21.4)	0.051
Lymphopenia*^θ^, *n (%)*	1 (6.3)	15 (34.9)	**0.045**
Persistent salivary gland swelling*^θ^, *n (%)*	0	8 (19.0)	0.092
Purpura/cutaneous vasculitis*^θ^, *n (%)*	1 (6.3)	8 (19.5)	0.420
Lymphoma			
Lymphoma*^∇^, *n (%)*	0	1 (3.3)	1.000
Non-Hodgkin lymphoma*^∇^, *n (%)*	0	0	NA
Baseline evaluation			
ESSDAI*^φ^, 0-123 (IQR)	7.0 (9.0)	10.0 (10.8)	0.577
ESSDAI without neurological domains*^φ^, 0-93 (IQR)	4.0 (7.0)	5.5 (8.0)	0.390
ESSPRI*^∇^, 0-10 (IQR)	2.7 (6.7)	5.7 (4.0)	0.292
Involvements (definitions according to ESSDAI score)			
Constitutional*^θ^, *n (%)*	3 (18.8)	11 (25.0)	0.740
Lymphadenopathic*^θ^, *n (%)*	4 (25.0)	5 (11.4)	0.230
Glandular*^θ^, *n (%)*	5 (31.3)	17 (38.6)	0.600
Articular*^θ^, *n (%)*	6 (37.5)	24 (54.5)	0.243
Cutaneous*^θ^, *n (%)*	5 (31.3)	13 (29.5)	1.000
Pulmonary*^θ^, *n (%)*	1 (6.3)	9 (20.5)	0.263
Renal*^θ^, *n (%)*	2 (12.5)	0	0.070
Muscular*^θ^, *n (%)*	0	0	NA
PNS, *n (%)*	0	45 (100)	NA
CNS, *n (%)*	16 (100)	0	NA
Haematological*^θ^, *n (%)*	7 (43.8)	17 (40.5)	0.821
Biological*^θ^, *n (%)*	10 (62.5)	27 (62.8)	0.984
Treatment (ever)			
Corticosteroids*^φ^, *n (%)*	9 (56.3)	24 (66.7)	0.472
Hydroxychloroquine*^φ^, *n (%)*	9 (56.3)	24 (66.7)	0.472
Pilocarpine*^φ^, *n (%)*	1 (6.3)	10 (27.8)	0.140
Methotrexate*^φ^, *n (%)*	3 (18.8)	7 (19.4)	1.000
Azathioprine*^φ^, *n (%)*	5 (31.3)	12 (33.3)	0.882
Leflunomide*^φ^, *n (%)*	0	2 (5.6)	1.000
Mycophenolate mofetil*^φ^, *n (%)*	0	3 (8.3)	0.544
Rituximab*^φ^, *n (%)*	4 (25.0)	6 (16.7)	0.475
Intravenous immunoglobulin*^φ^, *n (%)*	0	2 (5.6)	1.000
Cyclophosphamide*^φ^, *n (%)*	1 (6.3)	4 (11.1)	1.000
Death			
Death*^φ^, *n (%)*	0	3 (8.8)	0.543

Categorical variables are presented as frequency (percentage), and continuous variables as mean±standard deviation (SD) or median (interquartile range - IQR) for variables with skewed distributions. NA - not applicable.

θMissing data <10%; φ Missing data 10-25%; ∇ Missing data 25-50%; ◻Missing data >50%.

*SjD patients with CNS involvement - Race: n=15; Age at inclusion: n=7; Age at symptom onset: n=7; Age at diagnosis: n=7; Disease duration: n=7; Rheumatoid factor: n=15; Focus score≥1: n=11; Focus score: n=7; Ectopic lymphoid structures on salivary glands: n=9; Cryoglobulinaemia: n=11; Hypergammaglobulinaemia: n=15; Monoclonal gammopathy: n=15; Lymphoma: n=12; Non-Hodgkin lymphoma: n=12; ESSDAI: n=7; ESSDAI without neurological domains: n=7; ESSPRI: n=7; Death: n=15.

**SjD patients with PNS involvement - Race: n=37; Age at inclusion: n=10; Age at symptom onset: n=10; Age at diagnosis: n=10; Disease duration: n=10; AECG 2002: n=43; ANA: n=44; anti-SSA: n=44; anti-SSB: n=43; Rheumatoid factor: n=39; Focus score≥1: n=33; Focus score: n=10; Ectopic lymphoid structures on salivary glands: n=24; Cryoglobulinaemia: n=34; Hypergammaglobulinaemia: n=41; Monoclonal gammopathy: n=41; Low C3: n=42; Low C4: n=42; Lymphopenia: n=43; Persistent salivary gland swelling: n=42; Purpura/cutaneous vasculitis: n=41; Lymphoma: n=30; Non-Hodgkin lymphoma: n=30; ESSDAI: n=10; ESSDAI without neurological domains: n=10; ESSPRI: n=10; Constitutional: n=44; Lymphadenopathic: n=44; Glandular: n=44; Articular: n=44; Cutaneous: n=44; Pulmonary: n=44; Renal: n=43; Muscular: n=43; Haematological: n=42; Biological: n=43; Corticosteroids: n=36; Hydroxychloroquine: n=36; Pilocarpine: n=36; Methotrexate: n=36; Azathioprine: n=36; Leflunomide: n=36; Mycophenolate mofetil: n=36; Rituximab: n=36; Intravenous immunoglobulin: n=36; Cyclophosphamide: n=36; Death: n=32. Bold values indicate statistical significance (p<0.05).

Statistical analysis was performed with SPSS version 29.0.2.0 (IBM), and statistical significance was set at p<0.05.

### Ethics considerations

The study was conducted in accordance with the principles of the Declaration of Helsinki (as amended in 2024) and was approved by the Ethics Committee of Centro Académico de Medicina de Lisboa (CAML – 169/22) and the Reuma.pt National Committee. All included patients provided informed consent for Reuma.pt, allowing data collection for research use. Data were fully anonymised throughout the entire research process.

## Results

A total of 1375 patients with a diagnosis of SjD were screened, of whom 1234 had neurological involvement explicitly recorded as present or absent and were included. (**Supplementary Figure 1**) The demographic and clinical characteristics of the cohort are summarised in **Table 1**.

### Prevalence and characterisation of neurological involvement

Neurological involvement (PNS and/or CNS) was documented in 62 patients (5.0%, **Table 1**). Of these, 46 (3.7%) had PNS involvement, and 17 (1.4%) had CNS involvement. One patient presented both.

The proportion of female patients was lower in those with neurological involvement (n=54/62, 87.1% vs n=1108/1172, 94.5%, p=0.024). Ages at symptom onset, diagnosis and disease duration did not differ significantly between groups.

Clinical and laboratory markers of B-cell activity, including some associated with an increased risk for lymphoma, were more frequent in patients with neurological involvement. Cryoglobulinaemia was significantly more common in this group (n=7/46, 15.2% vs n=43/721, 6.0%, p=0.024), as were purpura/cutaneous vasculitis (n=9/58, 15.5% vs n=58/1037, 5.6%, p=0.007), and monoclonal gammopathy (n=7/57, 12.3% vs n=53/992, 5.3%, p=0.039). Other lymphoma risk factors, including lymphopenia (n=16/60, 26.7%), low C3 (n=11/59, 18.6%), low C4 (n=9/59, 15.3%), and persistent salivary gland swelling (n=8/59, 13.6%), were broadly similar between groups. One (n=1/42, 2.4%) patient with neurological disease presented a diagnosis of lymphoma.

Systemic disease activity was higher in patients with neurological involvement. Median ESSDAI was 6.0 (IQR 12.0) in the neurological cohort compared with 2.0 (IQR 4.0) in those without neurological disease (p<0.001). This difference persisted when neurological domains were excluded from the score [4.0 (IQR 7.0) vs 2.0 (IQR 4.0), p=0.002]. Most ESSDAI domains occurred at similar frequencies in both groups. However, cutaneous involvement (n=18/61, 29.5% vs n=201/1168, 17.2%, p=0.014) and pulmonary involvement (n=11/61, 18.0% vs n=90/1168, 7.7%, p=0.004) were significantly more frequent in the neurological cohort. In contrast, patient-reported symptom burden was comparable between the two groups [median EULAR Sjogren’s syndrome patient reported index (ESSPRI) (29) 5.8 (IQR 5.1) vs 5.5 (IQR 4.7), p=0.457].

Patients with neurological involvement more often received corticosteroids (n=34/53, 64.2% vs n=301/806, 37.3%, p<0.001) and immunosuppressive or biologic therapies, particularly azathioprine (n=18/53, 34.0% vs n=79/806, 9.8%, p<0.001), rituximab (n=10/53, 18.9% vs n=28/806, 3.5%, p<0.001), intravenous immunoglobulin (IVIg) (n=2/53, 3.8% vs n=4/806, 0.5%, p=0.048), and cyclophosphamide (n=5/53, 9.4% vs n=1/806, 0.1%, p<0.001). By contrast, hydroxychloroquine was prescribed less frequently in the neurological group (n=34/54, 64.2% vs n=639/806, 79.3%, p=0.010).

Mortality was higher among patients with neurological involvement (n=3/49, 6.1% vs n=4/639, 0.6%, p=0.010).

In the multivariable analysis, cryoglobulinaemia (OR 5.98, 95% CI 2.26-15.8, p<0.001) and a higher baseline ESSDAI, excluding the neurological domains, (OR 1.13, 95% CI 1.05-1.21, p<0.001) were identified as independently associated with neurological involvement. A sensitivity analysis restricted to patients fulfilling at least one set of classification criteria showed the same independent associations: cryoglobulinaemia (OR 6.58, 95% CI 2.10-20.6, p=0.001) and baseline ESSDAI, excluding the neurological domains (OR 1.10, 95% CI 1.01-1.19, p=0.032). In a sensitivity analysis, excluding cryoglobulinaemia (due to missing data), baseline ESSDAI, without the neurological domains, (OR 1.11, 95% CI 1.05-1.17, p<0.001) remained significant, and purpura/cutaneous vasculitis emerged as a further factor independently associated with neurological involvement (OR 2.66, 95% CI 1.19-5.99, p=0.018). (**Supplementary Table 1**).

### Peripheral nervous system involvement

**Figure 1:**
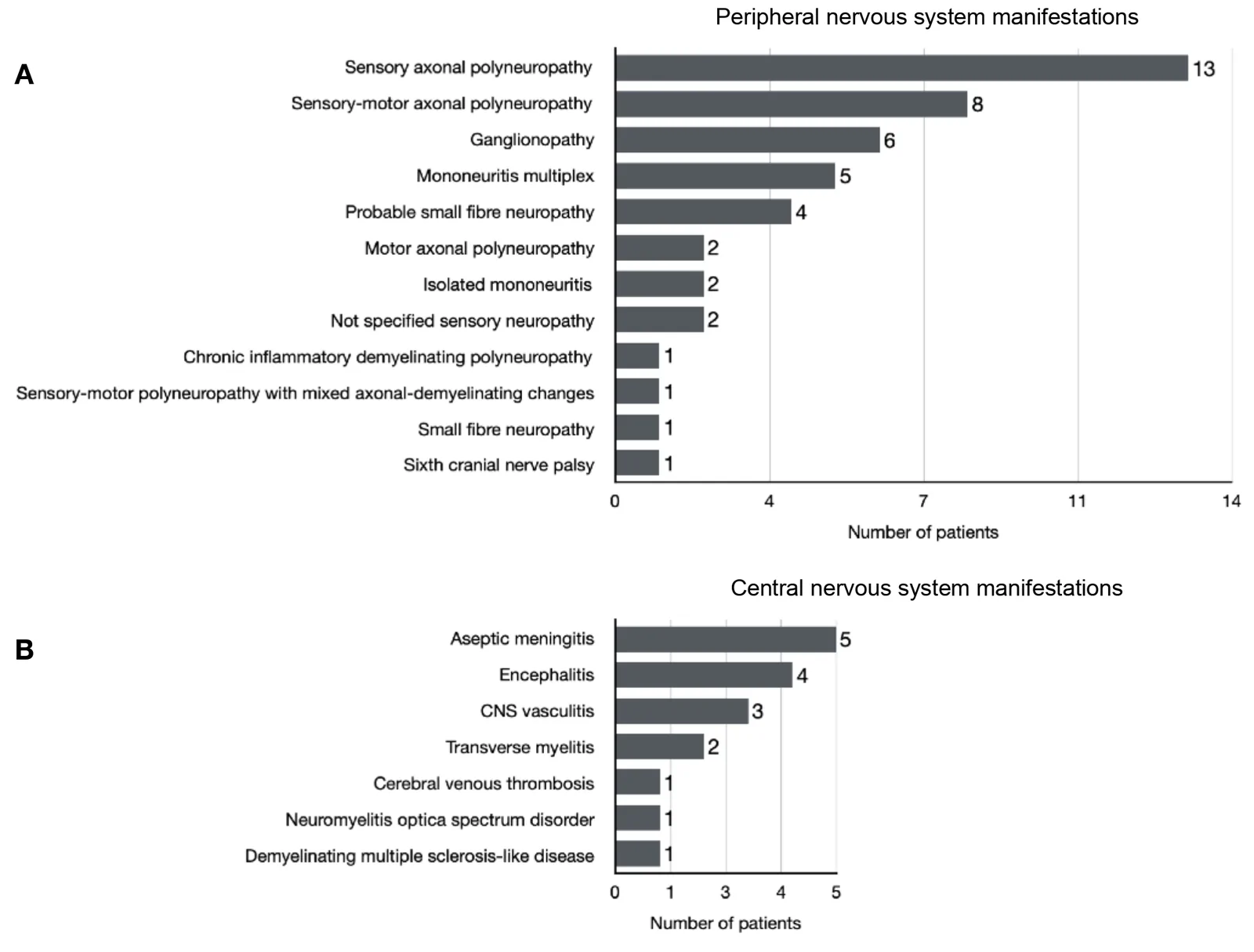
Distribution of PNS **(A)** and CNS **(B)** manifestations. Data are presented as absolute counts.

PNS involvement was present in 46 (3.7%) patients and consisted of sensory axonal polyneuropathy (n=13), sensory-motor axonal polyneuropathy (n=8), ganglionopathy (n=6), mononeuritis multiplex (n=5), motor axonal polyneuropathy (n=2), isolated mononeuritis (n=2), chronic inflammatory demyelinating polyneuropathy (n=1), sensory-motor polyneuropathy with mixed axonal-demyelinating changes (n=1), small fibre neuropathy (n=1), and sixth cranial nerve palsy (n=1). Of the remaining six patients, four had probable small fibre neuropathy (compatible symptoms with normal nerve conduction studies), whereas two were classified as having sensory neuropathy not otherwise specified, as nerve conduction studies were compatible but detailed data were unavailable. (**Figure 1A**).

With the exception of the patient with the cranial nerve palsy, all patients underwent electrophysiological studies, including nerve conduction studies and, when indicated, needle electromyography. Two patients with ganglionopathy performed somatosensory evoked potentials. The diagnosis of small fibre neuropathy was confirmed by quantitative sensory testing and skin biopsy.

PNS involvement was present at the time of SjD diagnosis in the majority of patients (n=22/41, 53.7%). In the remaining patients, the median time from diagnosis to neurological involvement was 3.9 (IQR 6.0) years.

In a sub-analysis comparing patients with and without PNS involvement (**Table 2**), the former were older at symptom onset (53.1 ± 13.7 vs 47.7 ± 14.8 years, p=0.024) and at diagnosis (57.2 ± 13.0 vs 52.7 ± 14.6 years, p=0.042).

Patients with PNS involvement more frequently exhibited clinical and biological markers associated with systemic and lymphoproliferative burden. Lymphopenia (n=15/44, 34.1% vs n=208/1052, 19.8%, p=0.021), low C4 (n=9/43, 20.9% vs n=89/1033, 8.6%, p=0.012), cryoglobulinaemia (n=7/35, 20.0% vs n=43/732, 5.9%, p=0.005), purpura/cutaneous vasculitis (n=8/42, 19.0% vs n=59/1053, 5.6%, p=0.003), and persistent major salivary gland swelling (n=8/43, 18.6% vs n=76/1059, 7.2%, p=0.013) were more frequent in the PNS group.

Systemic disease activity was higher in these patients, with a median ESSDAI of 9.0 (IQR 11.8) compared with 2.0 (IQR 4.0) in those without PNS disease (p<0.001). This difference persisted after exclusion of the neurological domains [4.0 (IQR 8.0) vs 2.0 (IQR 4.0), p=0.003]. Cutaneous and pulmonary ESSDAI domains were more frequently involved in the PNS group (n=13/45, 28.9% vs n=206/1184, 17.4%, p=0.048, and n=10/45, 22.2% vs n=91/1184, 7.7%, p=0.003, respectively), whereas other domains were broadly similar between groups.

Patients with PNS involvement more often received corticosteroids (n=25/37, 67.6% vs n=310/882, 37.7%, p<0.001) and immunosuppressive agents, including azathioprine (n=13/37, 35.1% vs n=84/822, 10.2%, p<0.001), rituximab (n=6/37, 16.2% vs n=32/822, 3.9%, p=0.004), IVIg (n=2/37, 5.4% vs n=4/822, 0.5%, p=0.024), and cyclophosphamide (n=4/37, 10.8% vs n=2/822, 0.2%, p<0.001). Mortality was also significantly higher in the PNS group (n=3/34, 8.8% vs n=4/654, 0.6%, p=0.003).

In the multivariable analysis, older age at symptom onset (OR 1.04, 95% CI 1.01-1.07, p=0.022), cryoglobulinaemia (OR 13.4, 95% CI 4.23-42.3, p<0.001), lymphopenia (OR 3.03, 95% CI 1.24-7.40, p=0.015), and baseline ESSDAI excluding the neurological domains (OR 1.12, 95% CI 1.02-1.23, p=0.016) emerged as factors independently associated with PNS involvement.

A sensitivity analysis restricted to patients fulfilling at least one set of classification criteria yielded similar findings, as age at symptom onset (OR 1.05, 95% CI 1.01-1.10, p=0.010), cryoglobulinaemia (OR 10.12, 95% CI 2.82-36.4, p<0.001) and lymphopenia (OR 4.04, 95% CI 1.48-10.99, p=0.006) were identified as associated factors. Of note, pulmonary involvement (OR 3.36, 95% CI 1.02-11.04, p=0.046) also emerged as an independent association.

A sensitivity analysis excluding cryoglobulinaemia reconfirmed age at symptom onset (OR 1.03, 95% CI 1.00-1.06, p=0.023) and baseline ESSDAI without the neurological domains (OR 1.09, 95% CI 1.02-1.17, p=0.018) as factors independently associated with PNS involvement. In addition, it identified an association of low C4 (OR 2.99, 95% CI 1.14-7.90, p=0.027), persistent salivary gland swelling (OR 3.50, 95% CI 1.38-8.91, p=0.008), and purpura/cutaneous vasculitis (OR 2.89, 95% CI 1.04-8.02, p=0.042) with this outcome. (**Supplementary Table 2**).

### Central nervous system involvement

CNS involvement was observed in 17 patients (1.4%). The manifestations were as follows: aseptic meningitis (n=5), encephalitis (n=4), CNS vasculitis (n=3), transverse myelitis (n=2), cerebral venous thrombosis (n=1), neuromyelitis optica spectrum disorder (n=1), and demyelinating multiple sclerosis-like disease (n=1). (**Figure 1B**).

Most patients underwent brain and/or spinal magnetic resonance imaging (n=11/14, 78.6%) and lumbar puncture (n=10/14, 71.4%). Other investigations included electroencephalogram (n=2), somatosensory evoked potentials (n=1), and brain positron emission tomography (n=1).

In most patients, CNS involvement was present at the time of SjD diagnosis (n=9/12, 75.0%). In the remaining patients, the mean time from diagnosis to neurological manifestations was 2.4 ± 3.0 years.

Compared with patients without CNS involvement, (**Table 3**) those with CNS manifestations were significantly younger at diagnosis (43.7 ± 16.4 vs 53.0 ± 14.5 years, p=0.014).

Systemic disease activity, assessed by the total ESSDAI score, was higher in patients with CNS involvement [4.5 (IQR 12.0) vs 2.0 (IQR 4.0), p=0.007]. However, this difference was no longer apparent when the neurological domains were excluded [2.5 (IQR 5.0) vs 2.0 (IQR 4.0), p=0.550]. There were no differences in the frequency of involvement of individual ESSDAI domains between the groups.

Azathioprine (n=6/17, 35.3% vs n=91/842, 10.8%, p=0.008) and rituximab (n=4/17, 23.5% vs n=34/842, 4.0%, p=0.005) were used significantly more often in patients with CNS disease.

In the exploratory multivariable analysis, male sex (n=3/17; OR 5.44, 95% CI 1.45-20.4, p=0.012) and a younger age at diagnosis (OR 1.05, 95% CI 1.01-1.08, p=0.009) were independently associated with CNS involvement. A sensitivity analysis restricted to patients fulfilling at least one set of classification criteria showed similar findings, as male sex (OR 5.19, 95% CI 1.03-26.14, p=0.046) and a younger age at diagnosis (OR 1.05, 95% CI 1.00-1.09, p=0.035) remained independently associated with this outcome. (**Supplementary Table 3**).

### Comparison of patients with PNS and patients with CNS involvement

A comparison between patients with PNS involvement and those with CNS involvement, excluding the single patient who had both, (**Table 4**) revealed that individuals with PNS manifestations were more often Caucasian (n=37/37, 100% vs n=12/15, 80.0%, p=0.021). These patients were also older at symptom onset (56.6 ± 11.7 vs 42.0 ± 10.6 years, p=0.017), at diagnosis (59.9 ± 12.8 vs 43.4 ± 10.3 years, p<0.001), and at neurological symptom onset (58.7 ± 13.7 vs 42.3 ± 14.6 years, p<0.001).

The immunological profile was broadly comparable between groups, except for anti-SSA antibodies which were less frequent in the PNS group (n=32/44, 72.7% vs n=16/16, 100%, p=0.025). Conversely, lymphopenia was more common in these patients (n=15/43, 34.9% vs n=1/16, 6.3%, p=0.045).

Systemic disease activity, overall disease burden, treatment exposure and mortality were comparable between groups.

Considering the small sample size, no multivariable analysis was performed.

## Discussion

In this large, nationwide real-world cohort of patients with SjD, neurological involvement was relatively infrequent (5.0%), with PNS involvement in 3.7% and CNS involvement in 1.4% of patients. These figures fall at the lower end of the wide range reported in previous studies, where overall neurological involvement has varied from 19.2 to 68% (7–13). Several methodological issues explain these discrepancies. Prior cohorts differ in classification criteria, inclusion of overlapping connective tissue diseases, sample size, and recruitment setting, which has ranged from Rheumatology and Internal Medicine to Neurology or mixed environments. Definitions of neurological involvement have also varied, particularly for CNS manifestations. Some studies included non-specific symptoms or MRI findings, while others systematically screened for subclinical neuropathy using electrophysiology. Conversely, more stringent studies included only clinically overt neurological manifestations supported by objective findings (4–17, 20, 22–26).

In this study, neurological involvement was defined by the treating physician in collaboration with a neurologist, and confirmed by detailed chart review. Inclusion required compatible clinical manifestations corroborated by diagnostic investigations and we did not include isolated non-specific symptoms or abnormal test results. We did not consider pure autonomic involvement, given the lack of standardisation in diagnostic criteria (18). This conservative approach, including the exclusion of isolated autonomic involvement and the requirement of clinically overt neurological disease supported by objective findings, likely contributed to the lower prevalence observed. In addition, the Rheumatology-based nature of the registry may have led to under-representation of patients presenting primarily to Neurology departments. Nevertheless, neurological involvement in this cohort identified a clinically meaningful subset of patients with a distinct profile.

Although our neurological cohort remained predominantly female, the proportion of men was significantly higher compared with the non-neurological group, consistent with previous observations (8, 12). Importantly, patients with neurological disease exhibited a distinct profile, characterised by higher frequencies of cryoglobulinaemia, purpura/cutaneous vasculitis, and monoclonal gammopathy, alongside higher systemic disease activity, even after exclusion of the neurological domains from the ESSDAI. Respiratory and cutaneous involvements were notably more common in this group. However, higher systemic activity did not translate into greater symptom burden, as reflected by similar ESSPRI scores, reinforcing previous findings that systemic inflammation and symptoms correlate poorly in SjD (3). Consistent with this more severe phenotype, patients with neurological involvement more frequently required corticosteroids and immunosuppressive therapy, and had higher mortality.

In the multivariable analysis, higher baseline ESSDAI (excluding neurological domains) was consistently identified as an independent association with neurological manifestations. Cryoglobulinaemia and purpura/cutaneous vasculitis also emerged as important associations, aligning with reports implicating vasculitic and B-cell-mediated mechanisms in the development of neurological complications (4). Collectively, these findings support that neurological involvement reflects a high-risk systemic phenotype rather than an organ-limited complication, with the need for intensive immunosuppression and worse prognosis (4–8, 10, 23).

As in most previous cohorts, PNS involvement was the most frequent neurological manifestation, predominantly presenting as sensory or sensory-motor axonal polyneuropathy (2, 5–8, 10, 15, 16, 24–26). Patients with PNS involvement displayed a distinct clinical and immunological profile. Older age at symptom onset, cryoglobulinaemia, lymphopenia, and higher baseline ESSDAI (excluding neurological domains) were independently associated with PNS manifestations, with pulmonary involvement emerging as an additional association in a sensitivity analysis including only patients who fulfilled classification criteria. When cryoglobulinaemia was removed due to missing data, low C4, persistent salivary gland swelling, and purpura/cutaneous vasculitis were also identified as associated factors. These findings align with prior studies linking PNS manifestations to systemic activity, hypocomplementaemia, and markers of B-cell activation, supporting a vasculitic, B-cell-driven pathophysiology underlying PNS involvement in SjD (4–6, 9, 22, 23). In addition, they echo large cohort studies identifying cryoglobulinaemia and hypocomplementaemia as major prognostic markers linked to systemic involvement and adverse outcomes (5, 10, 16). Our results extend these observations by demonstrating that this high-risk immunological profile is enriched among patients with PNS involvement and is independently associated with its occurrence. Furthermore, our findings support observations that PNS involvement increases with age (30). Clinically, these results identify a subgroup of older patients with high systemic activity and markers of vasculitic and lymphoproliferative risk who may warrant consideration of early neurological evaluation and closer clinical monitoring, with potential implications for therapeutic decision-making.

The spectrum of CNS manifestations in our cohort broadly overlapped with that reported in other cohorts (9, 10, 19, 20). Although total ESSDAI was higher in patients with CNS involvement, this difference disappeared when the neurological domains were removed. No clear pattern emerged in other systemic or laboratory markers and CNS disease was not associated with increased mortality, although the small sample size limits firm conclusions. In the multivariable analysis, male sex and younger age at diagnosis were independently associated with CNS involvement, while previously reported associations with hypocomplementaemia, anti-SSA and lung involvement were not confirmed (9, 11, 19).

Direct comparison of PNS and CNS subgroups further supports their distinctiveness as patients with PNS disease were older at symptom onset, at SjD diagnosis, and at the onset of neurological symptoms. They were also more frequently Caucasian and lymphopenic and less frequently anti-SSA positive.

Altogether, our findings are compatible with the notion that PNS and CNS involvement, although both included within the “neurological” domain, may reflect at least partially distinct disease pathways. This mirrors observations from other cohorts in which predictors of PNS and CNS involvement differed and supports the need to consider these entities separately in both clinical practice and research (4–6, 8, 9, 11, 13, 19, 22, 23).

In our cohort, neurological manifestations frequently preceded or coincided with the diagnosis of SjD, particularly in the CNS subgroup, as described in several other series (10, 11, 19, 20, 24). This reinforces that neurological disease may be a presenting feature of SjD, underscoring the need for neurologists to maintain a high index of suspicion in patients with unexplained inflammatory neuropathies or CNS syndromes. Given this frequent temporal overlap, and the fact that the baseline date corresponded to the first available registry entry, clinical and disease activity data may have temporally coincided with the neurological manifestations. Therefore, the findings of this study should be interpreted as independent associations rather than as causal factors.

Recent studies in juvenile SjD support the concept that age at disease onset may influence both clinical presentation and diagnostic recognition, with less prominent sicca symptoms and more frequent systemic features (31–33). Although these data provide broader context for age-related differences in SjD, they should not be interpreted as directly explaining the age associations observed in our cohort.

The main strengths of this study are the use of a large, nationwide, real-world registry; the inclusion of patients followed in specialised Rheumatology centres; and the detailed chart-verified characterisation of neurological involvement. By analysing the overall cohort of patients with neurological involvement and then evaluating PNS and CNS manifestations separately, we were able to show that these subgroups exhibit distinct clinical profiles and independent associations. This raises the question of whether PNS and CNS manifestations should be considered as a single, homogeneous neurological domain in SjD, or rather as divergent entities with potentially different underlying pathophysiological mechanisms, risk factors, clinical courses, and outcomes. However, the comparison of these phenotypes is usually hampered by the small number of patients within each subgroup.

Limitations of this study include the retrospective design and potential for missing data, under-reporting, and variability in neurological assessment. Missing data were present for several variables, and complete-case analysis may have introduced selection bias if data were not missing completely at random. However, sensitivity analyses excluding variables with a high proportion of missing data showed similar findings. Neurological involvement was not systematically screened for, and autonomic involvement was not captured, likely contributing to underestimation of the true prevalence, particularly of milder or subclinical disease. Additionally, the lack of uniform diagnostic criteria across centres may have introduced some heterogeneity and potential misclassification of neurological manifestations. Despite this, and even though attribution of neurological manifestations to SjD is inherently challenging, the close relationship with Neurology departments in included centres may have helped minimize these limitations. Moreover, the use of backward selection for multivariable modelling may have increased the risk of overfitting, particularly given the relatively low number of events for some outcomes. Although variable selection was informed by clinical reasoning, these findings should be interpreted with caution and require validation in independent cohorts. Additionally, the small number of patients with CNS involvement limited statistical power and may have resulted in model instability. To minimise overfitting, the initial multivariable model was restricted to two variables. Nevertheless, the multivariable analysis for CNS involvement should be considered exploratory and interpreted with caution, as its findings require confirmation in larger cohorts. Some associations showed large odds ratios with wide confidence intervals, reflecting sparse data and limiting interpretation of the precise magnitude of effect. Therefore, the direction of association is more informative than the effect size estimate, particularly for the CNS model. Furthermore, a significant proportion of patients did not fulfil SjD classification criteria, which may introduce heterogeneity and limit comparability with other cohorts. However, this reflects real-world clinical practice, particularly in patients with early or predominantly extraglandular disease, such as neurological manifestations. Importantly, the majority of the PORTRESS registry patients still had objective features supporting the diagnosis, including sicca symptoms and/or ESSDAI-defined extraglandular involvement together with anti-SSA/Ro positivity or positive minor salivary gland biopsy (3). In addition, the inclusion of clinically diagnosed patients who do not fulfil classification criteria has been recognised as an unmet need in SjD research (3). Nonetheless, sensitivity analyses restricted to patients meeting classification criteria yielded overall consistent results, supporting the robustness of our findings. Finally, the Rheumatology departments-based nature of the registry may have under-represented patients with SjD that present initially to Neurologists.

In summary, neurological involvement in this large Portuguese SjD cohort was infrequent but clinically meaningful. PNS involvement clustered with a high-risk systemic phenotype characterised by older age at symptom onset, increased disease activity, and markers of vasculitic and B-cell-driven disease. Conversely, CNS involvement was rarer and showed a distinct profile, associated with male sex and younger age at diagnosis, without a clear enrichment in classical high-risk immunological markers. Clinically, these findings may help raise clinical awareness of neurological involvement in SjD patients with unexplained neurological symptoms and reinforce the importance of considering PNS and CNS manifestations separately in clinical assessment. In particular, vasculitic and B-cell-driven features may heighten suspicion for PNS involvement, whereas unexplained CNS syndromes may warrant consideration of SjD as part of the differential diagnosis, even in the absence of classical high-risk immunological markers. Prospective studies with standardised definitions and systematic neurological assessment are needed to validate these associations, clarify their clinical implications, and refine risk-stratification strategies.

## Data Availability

The original contributions presented in the study are included in the article/**Supplementary Material**. Further inquiries can be directed to the corresponding author.
