# Supplementary material for: NeuroPORTRESS: prevalence, clinical spectrum, and factors associated with neurological involvement in Sjögren’s disease
Source: Front Immunol. 2026 Aug 10;17:1791369. doi: 10.3389/fimmu.2026.1791369 (PMC13500490; doi:10.3389/fimmu.2026.1791369)
Supplement: Supplementary file 1 [file Image1.pdf]

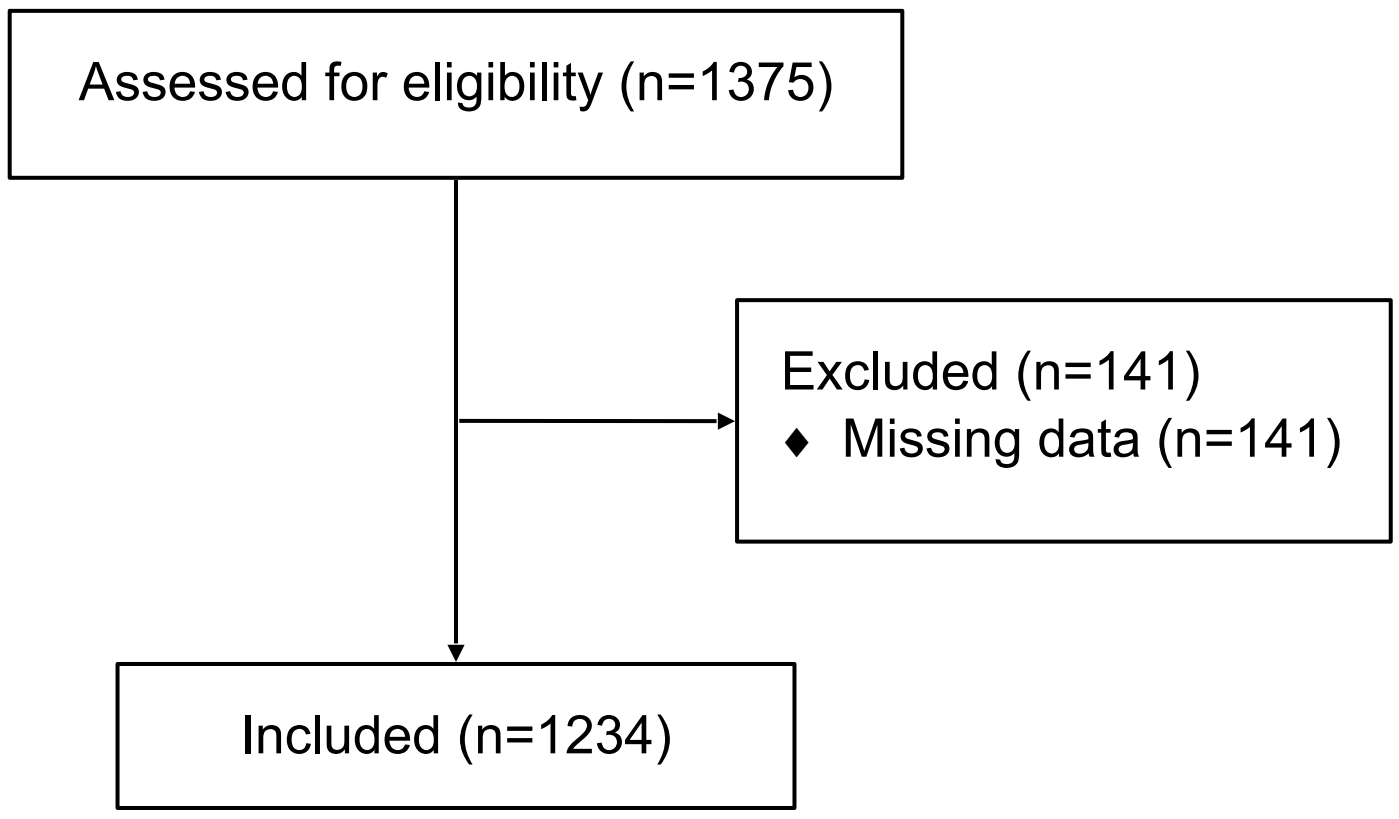

Supplementary Figure 1 - Flow diagram of patient selection.
