# Supplementary material for: NeuroPORTRESS: prevalence, clinical spectrum, and factors associated with neurological involvement in Sjögren’s disease
Source: Front Immunol. 2026 Aug 10;17:1791369. doi: 10.3389/fimmu.2026.1791369 (PMC13500490; doi:10.3389/fimmu.2026.1791369)
Supplement: Supplementary file 2 [file SupplementaryFile1.pdf]

Supplementary table 1.1 - Multivariable analysis - neurological involvement.

Variable	Multivariable model before backwards selection<br><br>OR (95% CI)<br>(N=579/1234)	Multivariable model before backwards selection<br><br>*p*-value	Multivariable model after backwards selection<br><br>OR (95% CI)<br><br>(N=649/1234)	Multivariable model after backwards selection<br><br>*p*-value
**Sex**	2.28 (0.81-6.49)	0.120	‡	‡
**Age at symptom onset**	1.00 (0.98-1.03)	0.842	‡	‡
**Cryoglobulinaemia**	**5.44 (1.72-17.2)**	**0.004**	**5.98 (2.26–15.8)**	**<0.001**
**Monoclonal gammopathy**	0.90 (0.23-3.61)	0.882	‡	‡
**Purpura/cutaneous vasculitis**	2.03 (0.71-5.82)	0.186	‡	‡
**Pulmonary involvement**	0.81 (0.26-2.59)	0.725	‡	‡
**Baseline ESSDAI excluding neurological domains**	**1.10 (1.02–1.20)**	**0.018**	**1.13 (1.05–1.21)**	**<0.001**

Variables selected for multivariable models: sex, age at symptom onset, cryoglobulinaemia, monoclonal gammopathy, purpura/cutaneous vasculitis, pulmonary involvement, baseline ESSDAI excluding neurological domains.

‡ Variables which were tested in the regression models but not retained after backward selection.

Supplementary table 1.2 - Multivariable analysis - neurological involvement, including only patients that fulfil at least one set of classification criteria.

Variable	Multivariable model before backwards selection<br><br>OR (95% CI)<br><br>(N=355/818)	Multivariable model before backwards selection<br><br>*p*-value	Multivariable model after backwards selection<br><br>OR (95% CI)<br><br>(N=481/818)	Multivariable model after backwards selection<br><br>*p*-value
**Sex**	3.38 (0.84-13.6)	0.085	‡	‡
**Age at symptom onset**	1.02 (0.99-1.05)	0.277	‡	‡
**Cryoglobulinaemia**	**12.8 (3.30-49.5)**	**<0.001**	**6.58 (2.10–20.6)**	**0.001**
**Monoclonal gammopathy**	1.94 (0.37-10.3)	0.436	‡	‡
**Purpura/cutaneous vasculitis**	0.69 (0.21-2.19)	0.524	‡	‡
**Pulmonary involvement**	1.31 (0.31-5.58)	0.711	‡	‡
**Baseline ESSDAI excluding neurological domains**	1.09 (0.98-1.22)	0.132	**1.10 (1.01–1.19)**	**0.032**

Variables selected for multivariable models: sex, age at symptom onset, cryoglobulinaemia, monoclonal gammopathy, purpura/cutaneous vasculitis, pulmonary involvement, baseline ESSDAI excluding neurological domains.

‡ Variables which were tested in the regression models but not retained after backward selection.

Supplementary table 1.3 - Multivariable analysis - neurological involvement, excluding cryoglobulinaemia.

**Variable**	**Multivariable model before backwards selection OR (95% CI) (N=835/1234)**	**Multivariable model before backwards selection *p*-value**	**Multivariable model after backwards selection OR (95% CI) (N=965/1234)**	**Multivariable model after backwards selection *p*-value**
**Sex**	2.21 (0.87-5.59)	0.095	‡	‡
**Age at symptom onset**	1.00 (0.98-1.01)	0.599	‡	‡
**Monoclonal gammopathy**	1.79 (0.63-5.11)	0.274	‡	‡
**Purpura/cutaneous vasculitis**	**2.47 (1.03-5.94)**	**0.043**	**2.66 (1.19-5.99)**	**0.018**
**Pulmonary involvement**	1.19 (0.47-3.06)	0.715	‡	‡
**Baseline ESSDAI excluding neurological domains**	**1.08 (1.01-1.16)**	**0.018**	**1.11 (1.05-1.17)**	**<0.001**

Variables selected for multivariable models: sex, age at symptom onset, monoclonal gammopathy, purpura/cutaneous vasculitis, pulmonary involvement, baseline ESSDAI excluding neurological domains.

‡ Variables which were tested in the regression models but not retained after backward selection.
