# Supplementary material for: NeuroPORTRESS: prevalence, clinical spectrum, and factors associated with neurological involvement in Sjögren’s disease
Source: Front Immunol. 2026 Aug 10;17:1791369. doi: 10.3389/fimmu.2026.1791369 (PMC13500490; doi:10.3389/fimmu.2026.1791369)
Supplement: Supplementary file 3 [file SupplementaryFile2.pdf]

Supplementary table 2.1 - Multivariable analysis - peripheral nervous system

Variable	Multivariable model before backwards selection<br><br>OR (95% CI)<br><br>(N=508/1234)	Multivariable model before backwards selection<br><br>*p*-value	Multivariable model after backwards selection<br><br>OR (95% CI)<br><br>(N=518/1234)	Multivariable model after backwards selection<br><br>*p*-value
**Sex**	2.05 (0.53-7.87)	0.297	‡	‡
**Age at symptom onset**	**1.04 (1.01-1.07)**	**0.024**	**1.04 (1.01-1.07)**	**0.022**
**Cryoglobulinaemia**	**11.4 (3.20-40.9)**	**<0.001**	**13.4 (4.23-42.3)**	**<0.001**
**Low C4**	1.44 (0.44-4.78)	0.549	‡	‡
**Lymphopenia**	**2.86 (1.11-7.41)**	**0.030**	**3.03 (1.24-7.40)**	**0.015**
**Persistent salivary gland swelling**	1.07 (0.28-4.08)	0.924	‡	‡
**Purpura/cutaneous vasculitis**	1.11 (0.41-3.03)	0.832	‡	‡
**Pulmonary involvement**	1.48 (0.40-5.42)	0.554	‡	‡
**Baseline ESSDAI excluding neurological domains**	**1.11 (1.01–1.23)**	**0.032**	**1.12 (1.02–1.23)**	**0.016**

Variables selected for multivariable models: sex, age at symptom onset, cryoglobulinaemia, low C4, lymphopenia, persistent salivary gland swelling, purpura/cutaneous vasculitis, pulmonary involvement, baseline ESSDAI excluding neurological domains.

‡ Variables which were tested in the regression models but not retained after backward selection.

Supplementary table 2.2 - Multivariable analysis - peripheral nervous system, including only patients that fulfil at least one set of classification criteria

Variable	Multivariable model before backwards selection<br><br>OR (95% CI)<br><br>(N=359/818)	Multivariable model before backwards selection<br><br>*p*-value	Multivariable model after backwards selection<br><br>OR (95% CI)<br><br>(N=424/818)	Multivariable model after backwards selection<br><br>*p*-value
**Sex**	3.20 (0.54-18.8)	0.198	‡	‡
**Age at symptom onset**	**1.05 (1.01-1.10)**	**0.029**	**1.05 (1.01-1.10)**	**0.010**
**Cryoglobulinaemia**	**18.1 (3.39-96.6)**	**<0.001**	**10.1 (2.82-36.4)**	**<0.001**
**Low C4**	1.51 (0.33-6.93)	0.593	‡	‡
**Lymphopenia**	**3.85 (1.20-12.4)**	**0.024**	**4.04 (1.48-11.0)**	**0.006**
**Persistent salivary gland swelling**	1.67 (0.34-8.13)	0.525	‡	‡
**Purpura/cutaneous vasculitis**	0.93 (0.25-3.42)	0.909	‡	‡
**Pulmonary involvement**	2.37 (0.51-11.1)	0.273	**3.36 (1.02-11.0)**	**0.046**
**Baseline ESSDAI excluding neurological domains**	1.04 (0.90-1.19)	0.628	‡	‡

Variables selected for multivariable models: sex, age at symptom onset, cryoglobulinaemia, low C4, lymphopenia, persistent salivary gland swelling, purpura/cutaneous vasculitis, pulmonary involvement, baseline ESSDAI excluding neurological domains.

‡ Variables which were tested in the regression models but not retained after backward selection.

Supplementary table 2.3 - Multivariable analysis - peripheral nervous system, excluding cryoglobulinaemia

Variable	Multivariable model before backwards selection<br><br>OR (95% CI)<br><br>(N=773/1234)	Multivariable model before backwards selection<br><br>*p*-value	Multivariable model after backwards selection<br><br>OR (95% CI)<br><br>(N=782/1234)	Multivariable model after backwards selection<br><br>*p*-value
**Sex**	1.39 (0.37-5.29)	0.627	‡	‡
**Age at symptom onset**	**1.03 (1.00-1.06)**	**0.032**	**1.03 (1.00-1.06)**	**0.023**
**Low C4**	2.49 (0.91-6.80)	0.076	**2.99 (1.14-7.90)**	**0.027**
**Lymphopenia**	1.94 (0.83-4.53)	0.125	‡	‡
**Persistent salivary gland swelling**	**3.45 (1.33-8.93)**	**0.011**	**3.50 (1.38-8.91)**	**0.008**
**Purpura/cutaneous vasculitis**	2.58 (0.89-7.48)	0.081	**2.89 (1.04-8.02)**	**0.042**
**Pulmonary involvement**	2.20 (0.80-6.09)	0.129	‡	‡
**Baseline ESSDAI excluding neurological domains**	1.06 (0.98-1.15)	0.143	**1.09 (1.02-1.17)**	**0.018**

Variables selected for multivariable models: sex, age at symptom onset, low C4, lymphopenia, persistent salivary gland swelling, purpura/cutaneous vasculitis, pulmonary involvement, baseline ESSDAI excluding neurological domains.

‡ Variables which were tested in the regression models but not retained after backward selection.
