# Supplementary material for: NeuroPORTRESS: prevalence, clinical spectrum, and factors associated with neurological involvement in Sjögren’s disease
Source: Front Immunol. 2026 Aug 10;17:1791369. doi: 10.3389/fimmu.2026.1791369 (PMC13500490; doi:10.3389/fimmu.2026.1791369)
Supplement: Supplementary file 4 [file SupplementaryFile3.pdf]

Supplementary table 3.1 - Multivariable analysis - central nervous system.

Variable	Multivariable model OR (95% CI) (N=1122/1234)	Multivariable model *p*-value
**Sex**	**5.44 (1.45-20.4)**	**0.012**
**Age at diagnosis**	**1.05 (1.01-1.08)**	**0.009**

Variables selected for multivariable models: sex, age at diagnosis.

Supplementary table 3.2 - Multivariable analysis - central nervous system, including only patients that fulfil at least one set of classification criteria.

Variable	Multivariable model OR (95% CI) (N=757/818)	Multivariable model *p*-value
**Sex**	**5.19 (1.03-26.1)**	**0.046**
**Age at diagnosis**	**1.05 (1.00-1.09)**	**0.035**

Variables selected for multivariable models: sex, age at diagnosis.
